# Cannabinoid actions on sensitized dural nociceptors in a non-surgical mouse model of migraine-like pain

**DOI:** 10.21203/rs.3.rs-8714966/v1

**Published:** 2026-03-03

**Authors:** Yoshihiro Kitaoka, Kyle Whyland, Zhiwei Li, Kyotaro Koshika, Toru Yamamoto, Yatendra Mulpuri, Igor Spigelman

**Affiliations:** University of California, Los Angeles; University of California, Los Angeles; University of California, Los Angeles; Tokyo Dental College; Niigata University; New York University; University of California, Los Angeles

**Keywords:** Acid-sensing ion channel, Cannabinoid, Dura mater, Migraine, Nociceptor sensitization, Trigeminal ganglion

## Abstract

**Background:**

Migraine is a debilitating neurological disorder affecting ~ 15% of the global population with greater prevalence in females. Cannabinoid 1 and 2 receptor (CB1R and CB2R) agonists alleviate migraine symptoms. However, central nervous system (CNS) side effects mediated by CB1Rs limit their widespread use. We developed peripherally restricted cannabinoids (PRCBs) which lack CNS side effects. Here we examine actions of a PRCB on behavioral and physiological parameters in a mouse migraine model.

**Methods:**

Female 4-week-old C57BL/6J mice were acclimated to measurements of head withdrawal threshold (HWT) responses to periorbital von Frey filaments. After several days of baseline testing, mice (under brief isoflurane anesthesia) were administered pH-6.0 or pH-7.4 saline (4.5μL) supradurally via a 33-gauge cannula through the skin and connective tissue by taking advantage of unfused cranial sutures at bregma, followed 3-days later, by responses to a mild (pH-7.0 saline) supradural stimulation. The CB1R/CB2R agonist PRCB, PrNMI (5μM), was co-applied with pH-6.0 saline with or without the peripherally-restricted selective CB1R antagonist, 18A (20μM) or the selective CB2R antagonist, SR144528 (20μM). The potent selective CB2R agonist RNB61 (5μM) was also co-applied. Patch clamp recordings were obtained in retrogradely-labeled dural trigeminal ganglion (dTG) neurons acutely isolated from mice treated 3-days earlier with supradural pH-7.4 or pH-6.0 saline and 4% fluorogold.

**Results:**

Supradural pH-6.0 (but not pH-7.4) treatment produced allodynia symptoms which recovered by 48-hrs but were renewed by pH-7.0 supradural treatment. Supradural co-administration of pH-6.0/PrNMI prevented both the initial and latent allodynia symptoms. Co-administration of pH-6.0/PrNMI with 18A, or SR144528, abolished the preventative effects of PrNMI. Co-administration of pH-6.0/RNB61 did not prevent the initial allodynia or latent sensitization. Co-administration of pH-7.0/PrNMI did not prevent latent hypersensitivity. All dTGs had Aδ- or C-type nociceptor characteristics. Acid-induced depolarizations were enhanced in dTGs from pH-6.0-treated mice. PrNMI (1μM) decreased acid-induced depolarizations in dTGs from pH-7.4- (but not pH-6.0)-treated mice. RNB61 (0.5μM) had no effect on acid-induced depolarizations in dTGs from either group.

**Conclusions:**

These findings indicate that PrNMI prevents acid-induced sensitization and allodynia by activating peripheral neuronal CB1Rs and non-neuronal CB2Rs. CB2R activation alone is insufficient, and once sensitized, combined CB1R/CB2R activation loses efficacy.

## INTRODUCTION

Migraine is a highly prevalent and disabling neurological disorder, affecting approximately 15% of the global population, with a disproportionately higher incidence observed among females. It is characterized by recurrent attacks of moderate to severe headache, often unilateral, and frequently accompanied by nausea, vomiting, photophobia, and phonophobia [1, 2]. Despite its substantial societal and individual burden, the precise pathophysiological mechanisms underlying migraine remain only partially understood, and existing therapeutic options frequently provide incomplete relief or are associated with adverse effects [3]. The most widely hypothesized mechanism underlying migraine pain involves the sensitization of meningeal sensory afferents by calcitonin gene-related peptide (CGRP) or nitric oxide (NO) donors [4, 5, 6]. This peripheral sensitization can propagate to second- and third-order neurons in the spinal trigeminal nucleus caudalis and the thalamus, respectively, leading to headache as well as cutaneous allodynia in both cephalic and extracephalic regions.

Cannabinoids acting on Gi/o-coupled cannabinoid 1 and 2 receptors (CB1Rs and CB2Rs) alleviate migraine-like pain symptoms in humans and animal models [7, 8, 9]. However, adverse effects mediated by CB1Rs in the central nervous system (CNS) limit their widespread use. Previous studies with conditional knockouts of peripheral CB1Rs showed that cannabinoid analgesia in inflammatory and neuropathic pain states is mediated largely by peripheral CB1Rs in nociceptors [10]. We developed synthetic peripherally restricted cannabinoid (PRCB) agonists that do not cross the blood-brain barrier, including 4-{2-[(1E)-1-[(4-propylnaphthalen-1-yl) methylidene]-1H-inden-3-yl]ethyl} morpholine (PrNMI) which effectively suppressed chronic pain symptoms in preclinical models of and traumatic nerve injury-, cancer, and chemotherapy- induced neuropathies, with minimal CNS-mediated side effects or development of tolerance [11, 12, 13]. Moreover, we demonstrated that PrNMI exerts prophylactic analgesic effects in mouse models of acute and chronic migraine-like pain induced by nitroglycerin, as well as sumatriptan-induced medication overuse headache [14]. However, a disadvantage of the nitroglycerin migraine model is that the high-dose nitroglycerin injection causes various adverse systemic changes, such as cardiovascular effects, not related to hyperalgesia, as well as widespread nociceptive effects not related to migraine. To address the shortcomings of the nitroglycerin model, we turned to a model of non-surgical dural stimulation which takes advantage of incomplete cranial suture fusion at bregma and lambda of young mice, allowing for deposition of fluids on the surface of the dura mater without surgery [15]. It also permits examination of hyperalgesic priming mechanisms focused on the trigeminal dural afferent neurons. Here, we used this model to examine the mechanisms of trigeminal dural afferent sensitization and the utility of PrNMI as a pre- and post-treatment in suppressing the behavioral symptoms of this sensitization.

## MATERIALS AND METHODS

### Animals

Female C57BL/6J mice (Jackson, Bar Harbor, ME) 4-weeks-old when delivered to UCLA were used. All mice were group housed (3–4 per cage) to avoid social isolation stress, in the vivarium under a 12-hr light/dark cycle (light on at 6 AM) and had *ad libitum* access to food and water during the entire experiment. All experiments were performed in accordance with the guidance of the National Institute of Health for the handling and use of laboratory animals and with approval from the Animal Research Committee of the University of California, Los Angeles.

### Behavioral testing

Prior to experiments, mice were randomly assigned to groups. All behavioral experiments were performed between 9 a.m. and 6 p.m. Investigators involved in behavioral testing, electrophysiological recordings, and data analysis were unaware of the nature and dose of administered experimental drugs.

### Assessment of mechanical sensitivity

Mice were initially acclimated to the testing environment for 2 hrs daily on 3 consecutive days. For periorbital sensitivity testing, mice were placed in 89-ml paper cups and placed in 5 cm length × 7.6 cm width × 15 cm height Plexiglas container with a 0.8 mm thick wire mesh (0.6 cm wide vertical slits) side, with the cup opening facing the mesh. Head withdrawal thresholds to von Frey filaments (Touch Test Sensory Evaluators, North Coast, Morgan Hill, CA, USA; bending force ranging from 0.008 to 2 g) applied to the periorbital region were determined after acclimating the mice in test cages for 15 minutes [14]. Measurements were performed by an investigator in a soundproofed room, at 300 lux soft white, fluorescent illumination. Individual von Frey filaments were applied to the periorbital region until it buckled slightly and held for several seconds or until a withdrawal occurred, termed positive responses. If a response was not noted, a denser filament test was performed; in the presence of a response, a lighter filament test was performed. Withdrawal thresholds were measured at 1-, 3-, 5-, and 24-hours post-injection using the up-down method [16, 17]. The maximum filaments used were 1 g for the periorbital region. Mice received 3 trials, and the average was taken of the 3 trials. Each trial was tested at least 5 minutes apart to prevent sensitization to the testing filaments. All criteria for the behavioral testing were determined *a priori* and followed throughout the study.

### Drugs

PrNMI was synthesized by Dr. Herbert H. Seltzman at the Research Triangle Institute (RTI, Research Triangle Park, NC) [12]. The peripherally-restricted rimonabant analog, 18A, was synthesized by Dr. Alan Fulp at RTI [18]. The CB1R and CB2R selective inverse agonists SR141716 (rimonabant) and SR144528, respectively were from the NIDA Drug Supply Program (National Institutes of Health, Bethesda, MD). RNB61 was from Tocris (Tocris Bioscience, Biotechne, Minneapolis, MN). Stock solutions of all drugs were prepared in dimethyl sulfoxide (DMSO) and stored at −20°C until use after appropriate dilution in various solutions. Solutions for supradural administration were composed of (in mM): NaCl 135, KCl 5, CaCl_2_ 2, MgCl_2_ 2, 4-(2-hydroxyethyl)-1-piperazineethanesulfonic acid (HEPES) 5, 2-(n-morpholino)-ethanesulfonic acid 5, sucrose 10. Solutions at pH 6.0, 7.0, and 7.4 were prepared by titrating the above solution with 1 M NaOH to reach the desired pH. Fluorogold (Fluorochrome LLC, Denver, CO) was dissolved in the pH 6.0 or 7.0 solution to 4%.

### Migraine-like pain mouse model

The acid-induced migraine-like pain model was adapted from [15]. Animals were tested for baseline responses 2–3 times before supradural administration of drugs. Supradural injectors were created by modifying commercially available cannulae (33-gauge, Part #8IC315I/SPC, Plastics One, Roanoke, VA). The projection depth of the internal cannula was set at 0.4 mm. The modified injector was attached to a 50 μL glass syringe needle (1705 series, Hamilton Company, Reno, NV) with Tygon^®^ tubing. Mice were anesthetized with an isoflurane vaporizer via nose cone, placed on a recirculating water heated blanket (HTP-1500, Kent Scientific, Torrington, CT) and injected through the skin and a cranial suture opening at bregma supradurally. After each injection, mice were monitored for full recovery from anesthesia and to ensure that no dura or brain damage was caused by the injection, and the animals were then returned to their home cages prior to subsequent behavioral testing.

### Acute TG neuron dissociation

The left and right trigeminal ganglia were dissected 3 days post-application of fluorogold to the dura mater. Acutely dissociated TG neurons from female C57BL/6J mice were prepared for recording as described previously [14], with modifications. Briefly, the excised TGs were placed into cold (4°C) modified Tyrode’s solution containing (in mM): NaCl 130, NaHCO_3_ 20, KCl 3, CaCl_2_ 4, MgCl_2_ 1, HEPES 10, glucose 12 and antibiotic/antimycotic solution (0.5%; Fisher Scientific, Hanover Park, IL). TG tissues were incubated in collagenase II (Invitrogen) and dispase II (Sigma-Aldrich) 1 mg/mL each for 1 hour at 37°C. TG tissues were washed twice with the modified Tyrode’s solution and triturated gently using fire-polished Pasteur glass pipettes. The cell suspension was mixed with bovine serum albumin (15%; Fisher Scientific), Scientific), centrifuged at 900 rpm for 10 minutes, the pellet was resuspended with Neurobasal A medium containing B27 (2%), L-glutamine (0.2%), and antibiotic/antimycotic solution (0.1%), and cells plated onto glass coverslips pre-coated with poly-D-lysine/laminin (all from Fisher Scientific), incubated at 37°C in a humidified 5% CO_2_ chamber and used within 8 hours after plating.

### Whole-cell patch clamp recordings

For most recordings, dissociated cells were incubated for 5 min with 10 μg/ml of fluorescein isothiocyanate (FITC)-conjugated isolectin B4 (IB4, Vector Laboratories, Burlingame, CA) in the external solution with the following composition (in mM): NaCl 140, KCl 4, CaCl_2_ 2, MgCl_2_ 2, HEPES 10, glucose 10, pH 7.4. IB4 positive neurons were visually identified using an FITC filter (excitation 465–495 nm; emission barrier filter 515–555 nm, Chroma Technology, Bellows Falls, VT) [19]. A cell was considered IB4 + ve if it had a continuous green ring around the perimeter at 10× magnification. Fluorogold positive cells were identified by their characteristic punctate cytoplasmic fluorescence at 40× magnification using a Fluorogold filter (excitation 350–420 nm; emission barrier filter 555–597 nm, Chroma Technology). The recording chamber was continuously perfused with external solution at a constant rate of 3 ml/min using a peristaltic perfusion system (Ismatec Reglo ISM-1B, Cole Palmer, Vernon Hills, IL). All external solutions were made fresh daily. On the day of the experiment, the compounds were diluted to their final concentrations in the external solution immediately before application. Patch electrodes were pulled from 1.5 mm OD borosilicate glass (Warner Instruments) on a horizontal puller (P-87, Sutter Instruments, Novato, CA) and had 3–10 MΩ resistances when filled with internal solution. Recordings were obtained in whole-cell configuration from TG neurons positive for intracellular fluorogold fluorescence. Electrophysiological data were acquired with patch electrodes placed in ISO-S-1.5G holders (G23 Instruments, London, UK) attached to the CV-7B head stage of a MultiClamp700B amplifier, Digidata 1400A, and using pCLAMP 10 software (Molecular Devices) running on a desktop computer (Dell Technologies, Austin TX) with a Windows 10 operating system (Microsoft, Redmond, WA). Following attainment of whole-cell access, recordings were initiated only after a 2–3 min period to ensure cellular equilibration. Series resistance (Rs) was evaluated by analyzing the decay time constant of the capacitance transients (uncompensated Rs less than 15 MΩ). Rs was monitored and compensated during the course of experiments. The liquid junction potential associated with all test solutions was less than 5 mV and therefore not corrected. Whole-cell capacitance was compensated with amplifier circuitry. Acid-induced responses were evoked either by bath perfusion or by fast switching to gravity-fed external solutions (with pH adjusted to 6.0 with 1 M HCl) with a computer-controlled fast-switching 3-barreled pipette (700 μm inside barrel diameter) placed 200 μm away from the target cell (SF-77B, Warner Instruments, Hamden, CT). PrNMI (1 μM) and RNB61 (0.5 μM) were bath-applied to determine their effect on acid-induced responses in TG neurons. All recordings were conducted at room temperature. Cell images were acquired using a monochrome digital camera (Basler) and processed with Adobe Photoshop 2025 (Adobe, San Jose, CA) software. Only cells with resting membrane potential more negative than − 40 mV were used for analysis.

### Statistical analysis

Data were graphed as mean ± SEM, The Shapiro-Wilk test was performed to check data normally. For two-group comparisons, unpaired Student’s *t*-test was performed. For multiple comparisons, one-way and two-way analysis of variance (ANOVA) or repeated measures ANOVA followed by the appropriate post-hoc test using Prism 10 software (GraphPad Software, La Jolla, CA). The value of P < 0.05 was considered statistically significant. Statistical analysis for individual experiments is described in figure legends.

## RESULTS

### Co-treatment with PrNMI prevents acid-induced allodynia and latent sensitization by activating CB1 and CB2 receptors.

**Figure 1:**
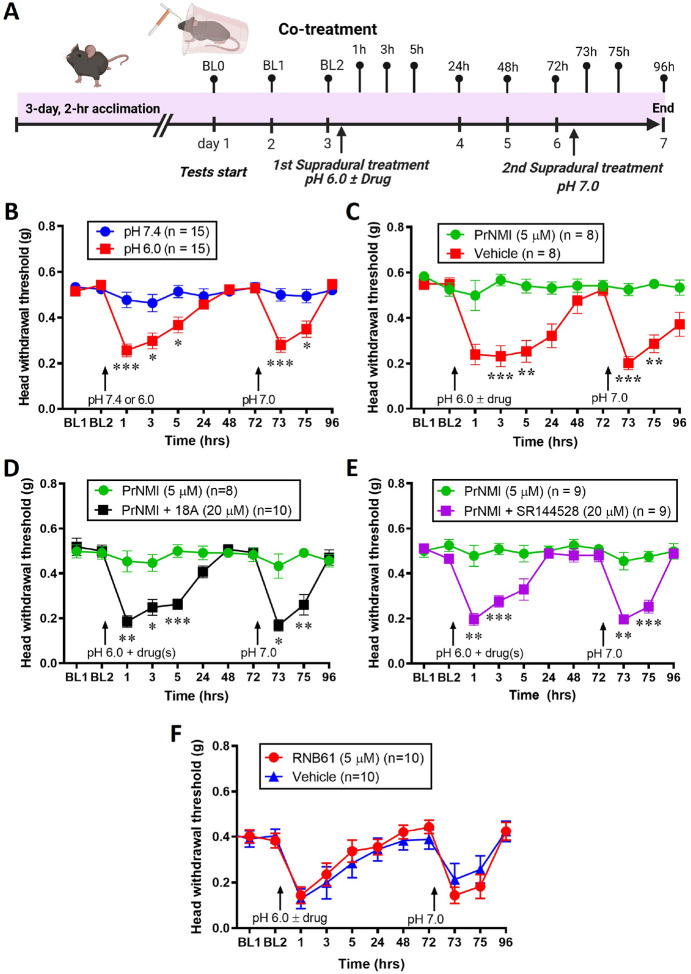
Co-treatment with PrNMI prevents acid-induced allodynia and latent sensitization by activating CB1 and CB2 receptors. A: Schematic representation of the experiment. B: Supradural administration of pH-6.0 but not pH-7.4 saline produces allodynia in the periorbital area which recovers by 48 hrs. At 72 hrs, supradural administration of pH-7.0 saline induces allodynia only in mice previously injected with pH-6.0 saline. C: PrNMI co-administration with pH-6.0 saline prevents both episodes of allodynia. D: Analgesic effects of PrNMI are blocked by co-administration with the peripherally-restricted selective CB1R inverse agonist, 18A. E: Analgesic effects of PrNMI are blocked by co-administration with the selective CB2R inverse agonist, SR144528. * P < 0.05, ** P < 0.01, *** P < 0.001 (two-way repeated measures analysis of variance). F: Co-administration of RNB61 with pH-6.0 saline does not prevent the initial or latent allodynic responses. P = 0.9326 (two-way repeated measures analysis of variance).

We first examined behavior responses in groups of mice that received supradural injections of either pH 6.0 or pH 7.4 (Fig. 1A). Mice that received an initial injection of a pH 6.0 solution showed significant decreases in head withdrawal thresholds to periorbital mechanical stimulation with von Frey hairs at 1, 3, and 5 hours, returning gradually to baseline by 48 hours. A second supradural injection of a pH 7.0 solution, administered 72 hours later, again induced a significant reduction in threshold at 1 and 3 hours, which resolved by 24 hours. In contrast, mice initially injected with a pH 7.4 solution showed no change in head withdrawal thresholds following either the first or second injection. Thus, mice in the pH 6.0 group experienced two distinct episodes of periorbital hypersensitivity to mechanical stimuli, whereas no such episodes were observed in the pH 7.4 group (Fig. 1B). In contrast to the vehicle-treated group, which exhibited mechanical hypersensitivity following the first injection, co-administration of PrNMI (full agonist at CB1R and partial agonist at CB2R [12]) with a pH 6.0 solution during the initial injection prevented the occurrence of both the initial and the latent periorbital allodynia (Fig. 1C). Coadministration of PrNMI with pH 6.0 and 18A, a peripherally restricted selective CB1R inverse agonist, abolished the analgesic effect of PrNMI, resulting in the appearance of hypersensitivity episodes following both the first and the second injections (Fig. 1D). Co-administration of SR144528, a selective CB2 receptor inverse agonist, also abolished the analgesic effect of PrNMI (Fig. 1E).

### RNB61 cotreatment does not prevent acid-induced allodynia and latent sensitization.

Based on the involvement of CB2R in PrNMI’s analgesic effects, we next evaluated the analgesic efficacy of RNB61, a highly potent selective CB2 receptor agonist [20]. Thus, after baseline threshold tests, either vehicle or RNB61 (5 μM) was co-administered with the pH 6.0 solution during the initial supradural injection, and a second supradural injection of a pH 7.0 solution was administered 72 hours later (Fig. 1A). No significant differences in nociceptive responses were observed between RNB61-treated and vehicle-treated animals, either immediately following the first injection or those triggered by a pH 7.0 treatment at 72 hours (Fig. 1F).

### Post-treatment with PrNMI does not prevent latent allodynia.

**Figure 2:**
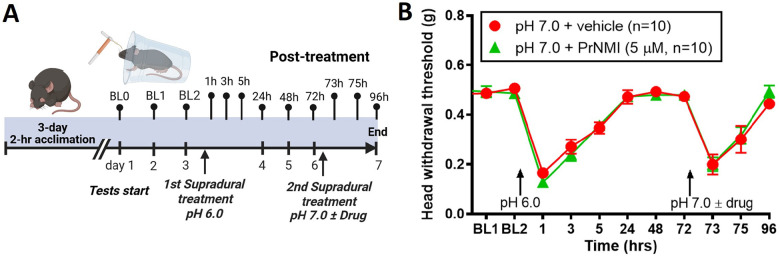
Post-treatment with PrNMI does not prevent latent allodynia. A: Schematic representation of experiment. B: Supradural treatment with pH-6.0 saline produces allodynia in the periorbital area which recovers by 24 hrs. At 72 hrs, supradural co-administration of PrNMI with pH-7.0 saline does not prevent latent allodynia responses. P = 0.9355 (two-way repeated measures analysis of variance).

Our previous studies with the nitroglycerin model of migraine-like pain in mice showed that while PrNMI was effective as a pre-treatment it failed to suppress periorbital allodynia as a post-treatment after repetitive nitroglycerin injections [14]. To examine PrNMI’s effectiveness as a post-treatment in the non-invasive supradural model, we administered a pH 6.0 solution as the initial injection in two groups of mice to induce initial allodynia and latent sensitization (Fig. 2A). Following the initial injection of the pH 6.0 solution, head withdrawal thresholds returned to baseline levels within 24 hours in both groups and at 72 hours, a second injection of a pH 7.0 solution in the control (vehicle) group evoked a latent sensitization-induced allodynia (Fig. 2B). However, when PrNMI was co-administered with a pH 7.0 solution as a post-treatment it did not alleviate this latent allodynia (Fig. 2B).

### Imaging of acutely dissociated dural TG neurons labeled with fluorogold and IB4.

**Figure 3:**
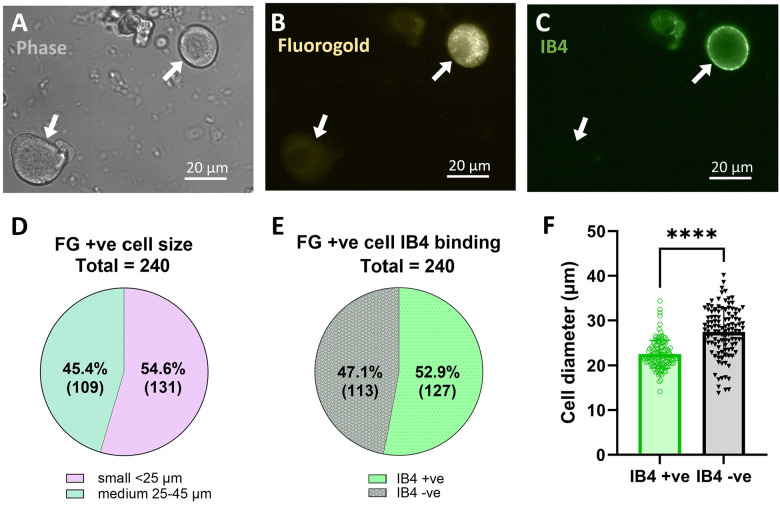
Acute dissociation of dural TG neurons labeled with Fluorogold and Isolectin B4. A: Phase contrast image of two acutely isolated TG neurons (arrows). B: Neuron at top right exhibits intracellular punctate fluorogold-positive fluorescence. C: Neuron at top right also exhibits IB4-positive membrane fluorescence. D: Distribution of fluorogold-positive (FG+ve) cell sizes. 62 mice. E: Ratio of FG+ve neuron is also positive for IB4 binding, which predominantly labels non-peptidergic nociceptors. A slight majority of FG+ve neurons were IB4 +ve (52.9%). F: Size comparison of IB4+ve and IB4-ve cells. **** P < 0.0001, (unpaired t-test t (238) = 8.907).

To study the cellular mechanisms of acid-induced sensitization of sensory neurons innervating the dura, mice received supradural injections of either pH 7.4 or pH 6.0 solutions concurrently with 4% fluorogold (FG). At 72 hours post-injection, trigeminal ganglia were acutely dissociated, placed in the recording chamber, and examined for the presence of FG labeled neurons as well as their IB4 binding (Fig. 3A–C). A total of 240 FG positive neurons were detected from a total of 124 ganglia, averaging ~ 2 FG +ve neurons/ganglion. FG +ve neurons were of small (< 25 μm) and medium (25–45 μm) size, with no large size (> 45 μm) FG +ve neurons detected (Fig. 3D). Majority were small-sized neurons, and more than half of the neurons were IB4 + ve (Fig. 3D, E). IB4 + ve neurons were significantly smaller in size compared to IB4 −ve neurons (Fig. 3F).

### Dural FG +ve TG neurons are Aδ- and C-type nociceptors.

**Figure 4:**
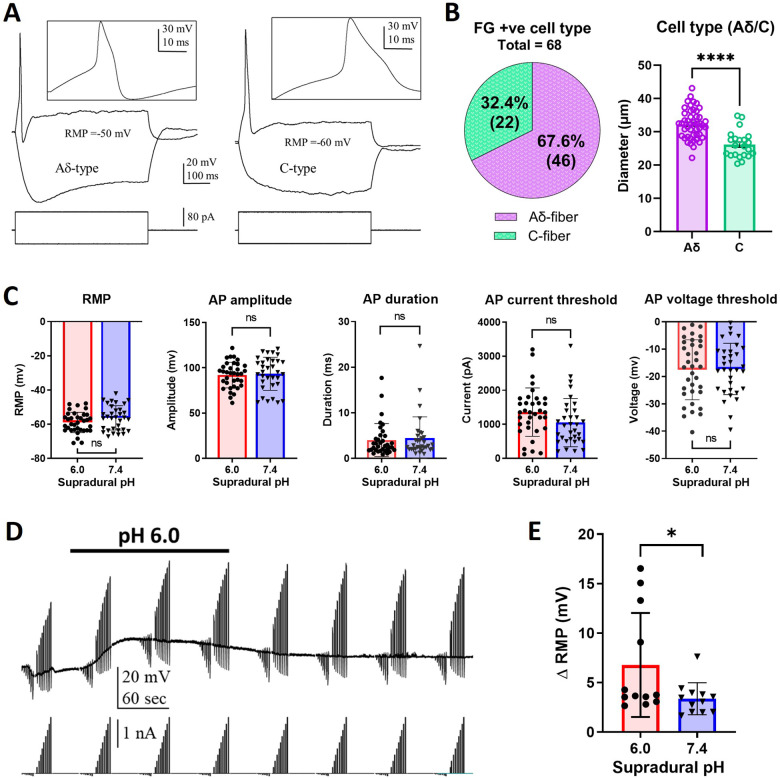
Dural FG+ve Aδ- and C-type nociceptors isolated from supradural acid-treated mice exhibit enhanced responses to acid. A: Representative voltage responses of FG+ve Ad- and C-type TG neurons to depolarizing and hyperpolarizing current steps. Both cell types exhibit action potentials with a hump on the repolarizing phase. In addition, Ad neurons exhibit a voltage sag in response to hyperpolarizing current pulses, whereas C-type neurons do not. B: Size distribution of FG+ve Ad- and C-type TG neurons. **** P < 0.0001, (unpaired t-test t (66) = 5.501). C: Resting membrane potential (RMP) and action potential (AP) properties of FG+ve TG neurons from supradural pH 6.0- and 7.4-treated mice. Resting membrane potential (RMP). P = 0.0993, (unpaired t-test t (66) = 1.672). AP amplitude. P = 0.7286, (unpaired t-test t (66) = 0.3484). AP duration. P = 0.6897 (unpaired t-test t (66) = 0.4010). AP current threshold. P = 0.08, (unpaired t-test t (66) = 1.795). AP voltage threshold. P = 0.9120, (unpaired t-test t (66) = 0.110). pH 7.4-treated mice = 26 mice. pH 6.0-treated mice = 19 mice. D: Voltage responses of an FG+ve Ad-type TG neuron to depolarizing and hyperpolarizing current steps before, during, and after a 3-min bath perfusion of pH-6.0. E: Graph of depolarizing responses to bath application of pH-6.0 in FG+ve TG neurons from supradural pH 6.0- and pH 7.4-treated mice. * P = 0.0434, (unpaired t-test t (22) = 2.144). pH-7.4 = 8 mice. pH-6.0 = 11 mice.

Patch-clamp recordings were made in acutely dissociated FG +ve TG neurons from mice that received supradural injections of either pH 7.4 or pH 6.0 solutions. Electrophysiological profiling identified FG +ve neurons with Aδ- and C- but not Aβ-type characteristics. Both Aδ- and C-type neurons exhibited a distinct hump during the action potential repolarization (Fig. 4A), unlike Aβ-type neurons (recorded from several FG −ve neurons, not shown). Aδ-neurons could be reliably distinguished from C-type neurons which lacked a voltage sag in response to hyperpolarizing current pulses (Fig. 4A). Notably, the average size of Aδ-type neurons was significantly larger than that of C-type neurons (Fig. 4B). Membrane properties of FG +ve neurons were evaluated following supradural treatment of mice with pH 7.4 or pH 6.0 solutions. (Fig. 4C). No statistically significant differences were observed between the two groups in their resting membrane potential or evoked action potential parameters (Fig. 4C).

### Enhancement of responses to acid in TG neurons from supradural acid-treated mice.

We next examined the responses of FG +ve TG neurons from mice that received supradural injections of either pH 7.4 or pH 6.0 solutions to bath application of pH 6.0 external solution (Fig. 4D). Twelve of 19 neurons from the pH 6.0-treated mice and 12 of 15 neurons from the pH 7.4-treated mice responded to bath application of pH 6.0 external solution with a membrane potential depolarization which we and others have previously shown to be mediated by activation of acid sensing ion channels (ASIC) in sensory neurons [21, 22, 14]. Analysis revealed that the magnitude of the pH 6.0-induced depolarization was significantly greater in FG +ve neurons from pH 6.0-treated mice than those from pH 7.4-treated controls (Fig. 4E).

### PrNMI decreases acid-induced depolarization in neurons from control but not acid-treated mice.

**Figure 5:**
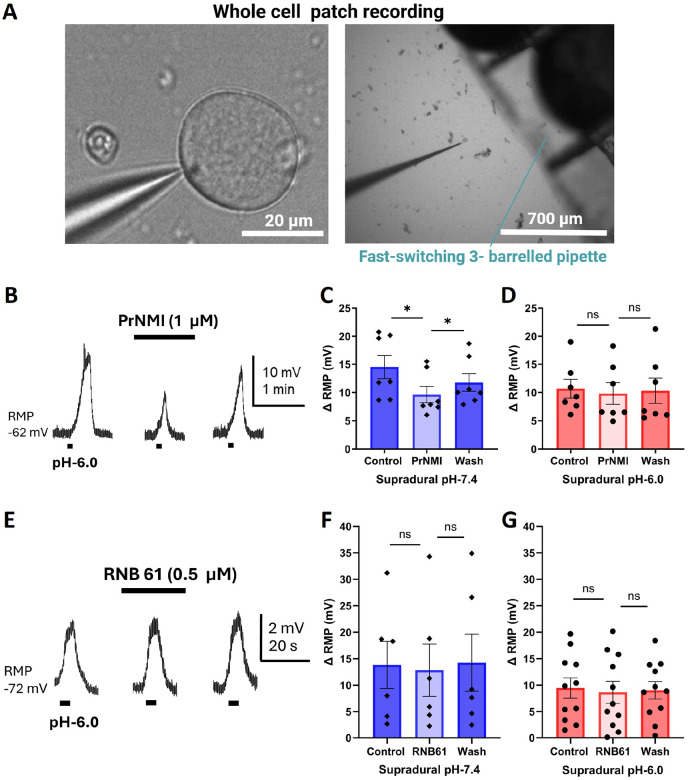
PrNMI decreases acid-induced depolarization in neurons isolated from control but not acid-treated mice. A: Schematic representation of fast switching perfusion experiment. B: PrNMI reversibly decreased the response to pH-6.0 of an FG+ve TG neuron from a supradural pH-7.4-treated mouse. C: Graph of FG+ve TG neuron depolarizing responses before, during, and after a 3-min bath perfusion of PrNMI from mice treated with supradural pH-7.4. * P = 0.0145, F (1.254, 7.525) = 9.177 (one-way repeated measures analysis of variance). pH7.4 = 9 mice. D: Graph of FG+ve TG neuron depolarizing responses before, during, and after a 3-min bath perfusion of PrNMI from mice treated with supradural pH-6.0. P = 0.6132, F (1.033, 6.199) = 0.2952 (one-way repeated measures analysis of variance). pH-6.0 = 6 mice. E: RNB61 did not decrease the response to pH-6.0 of an FG+ve Ad-type TG neuron from a supradural pH-7.4-treated mouse. F: Graph of FG+ve TG neuron depolarizing responses before, during, and after a 3-min bath perfusion of RNB61 from mice that received supradural pH-7.4 injections. P = 0.6227, F (1.285, 6.427) = 0.3585 (one-way repeated measures analysis of variance). pH-7.4 = 2 mice. G: Graph of FG+ve TG neuron depolarizing responses before, during, and after a 3-min bath perfusion of RNB61 from mice that received supradural pH-6.0 injections. P = 0.5870, F (1.539, 15.39) = 0.4822 (one-way repeated measures analysis of variance). pH-6.0 = 8 mice.

We next compared the effectiveness of a 3-minute bath application of PrNMI (1 μM) to suppress pH 6.0-induced depolarizations in FG +ve neurons isolated from pH 6.0- and pH 7.4-treated mice. In these experiments, we used a fast-switching 3-barreled pipette (Fig. 5A) to apply pH 6.0 external solution for 5-seconds at 5-minute intervals to minimize any ASIC-mediated response desensitization [14]. In neurons from pH 7.4-treated mice, PrNMI significantly suppressed the acid-evoked depolarizing potentials (Fig. 5B). There were significant differences in the amplitude of the responses to fast acid perfusion among the baseline, during the 3-minute PrNMI application, and after washout with control solution (Fig. 5C). In contrast, in the pH 6.0-treated group, PrNMI did not decrease the acid-evoked depolarizing potentials during the 3-minute application period (Fig. 5D).

### RNB61 does not decrease acid-induced depolarization in neurons from control or acid-treated mice.

To investigate the cellular effects of RNB61, a three-minute bath perfusion of the compound was applied to FG +ve TG neurons during patch-clamp recordings. Fast perfusion with a pH 6.0 solution was performed at 5-min intervals for 5-seconds before, during and after a 3-minute RNB61 exposure (Fig. 5E). In FG +ve TG neurons from either pH 7.4- or pH 6.0-treated mice, RNB61 failed to attenuate the depolarizing responses elicited by pH 6.0 stimulation (Fig. 5F, G).

## DISCUSSION

### Acid-induced acute allodynia and latent sensitization are prevented by co-treatment with PrNMI.

We used non-surgical supradural administration of an acidic (pH 6.0) solution in 5-week-old mice to induce acute allodynia which after full recovery at 3 days could be induced by a mild (pH 7.0) stimulus (Fig. 1). In the control group, no pain responses were observed following pH 7.0 treatment, suggesting that a threshold exists at pH 6.0 for activating dural afferents and eliciting nociceptive responses [15, 23, 24]. These results further suggest that despite recovery from migraine-like pain symptoms after acid stimulation, the initial reversible allodynia is associated with latent sensitization mediated by acid-sensing ion channels (ASICs) [25, 21]. We also demonstrate that co-treatment with the synthetic peripherally restricted cannabinoid agonist, PrNMI [12], suppressed the development of both acute allodynia and latent sensitization. The analgesic effects of PrNMI were prevented by cotreatment with a peripherally restricted CB1R inverse agonist, 18A [18], underscoring the importance of peripheral CB1R activation for analgesia (Fig. 1). Activation of CB1R has been shown to suppress CGRP release from TG neurons, the brainstem, and trigeminal sensory fibers innervating the dura [26, 27], as well as inhibit trigeminovascular brainstem neurons with A- and C-fiber input that innervate the dura mater [28]. However, PrNMI analgesia was also prevented by cotreatment with a selective CB2R inverse agonist, SR144528. Similar findings were reported by us [14] and others [27, 29] with the nitroglycerin-induced nociception model. For example, methanandamide, a brain-penetrant synthetic CB1R/CB2R agonist, was demonstrated to suppress CGRP release via CB1R and inhibit meningeal mast cell degranulation via CB2R [27].

### Selective activation of CB2R does not suppress acid-induced acute allodynia and latent sensitization or acid-induced depolarization in TG neurons

We also demonstrated that co-treatment with the potent, selective CB2R agonist, RNB61 [20] does not suppress the acid-induced allodynia and latent sensitization (Fig. 1F). CB2R are abundantly expressed in immune cells and glial cells and are implicated in the modulation of inflammatory and neuropathic pain [30, 31]. Various preclinical studies have shown that selective CB2R agonists suppress pain symptoms of inflammatory and neuropathic origin without psychotropic side effects, reviewed in [32, 33, 34, 30, 31]. However, successful translation of preclinical findings to humans has yet to materialize in part given the considerable divergence of human CB2R from the rodent orthologues, as well as an incomplete understanding of the mechanisms of CB2R-mediated suppression of pain symptoms, and the role of ligand-specific biased signaling in CB2R-mediated analgesia in various animal models of inflammatory and neuropathic pain [34, 30, 31, 35]. Early studies reported the absence of detectable CB2R mRNA in control rat DRG and TG sensory neurons [36, 37] or spinal cord [38] whereas expression could be induced in the spinal cord of neuropathic but not inflammatory chronic pain models [38]. Recently, RNAscope studies detected low levels of CB2R mRNA in DRG neurons and satellite glial cells and demonstrated increased mRNA expression after spinal nerve injury [39]. Other recent studies used conditional deletion of CB2R from peripheral sensory neurons to demonstrate loss of CB2-mediated antinociceptive efficacy in mouse models of inflammatory and neuropathic pain [40, 41]. In the present study, RNB61 also failed to suppress ASIC-mediated depolarizations in dural TG neurons isolated from either pH 7.4- or pH 6.0-treated mice (Fig. 5). This lack of RNB61 effectiveness points to the likely absence of functional CB2R in both control and sensitized dural TG neurons and hence a lack of direct impact on ASIC activity. Together, these findings indicate that selective activation of CB2R alone is unlikely to produce sufficient analgesic effects in this model of migraine-like pain. By contrast, PrNMI’s activation of TG neuron CB1R and concomitant activation of CB2R on immunocompetent cells contribute to the prevention of acid-induced acute allodynia and latent sensitization.

### PrNMI posttreatment does not prevent acid-induced latent sensitization.

The ineffectiveness of PrNMI as a posttreatment in the current study (Fig. 2) and in the chronic nitroglycerin model [14] contrasts with the primarily CB1R-mediated posttreatment effectiveness of PrNMI in suppression of chronic pain symptoms induced by peripheral nerve injury, bone cancer, and chemotherapy [11, 12, 13]. This is indicative of differences in migraine pathophysiology from that of other chronic pain states. Our exploration of the cellular mechanisms of acid-induced allodynia and sensitization revealed that all back-labelled dural sensory neurons were either Aδ- or C-type nociceptors (Figs. 3 and 4). Acidic stimulation is known to contribute to sensitization of the trigeminovascular system in migraine, reviewed in [21, 42]. In sensitized meningeal afferents, enhanced activation of ASIC channels facilitates the induction of action potentials even by subthreshold stimuli, thereby augmenting nociceptive signal transmission to the CNS [15]. Consistent with these previous reports, our data showed that TG neurons exposed to an acidic environment exhibited no major changes in membrane properties yet displayed significantly increased responses to acid stimulation (Fig. 4). Previous studies demonstrated that ASIC channels are downstream targets of potentiation by cyclic adenosine monophosphate (cAMP) produced by adenylyl cyclase [43, 44, 45]. Phosphorylation of protein kinase A (PKA) by cAMP also increases the activity of numerous other downstream targets such as voltage-gated Na^+^ [46] and Ca^2+^ [47, 48, 49] channels, enzymes such as nNOS [50, 51], and ligand-gated TRPA1 [52] and TRPV1 [53] channels. The increased magnitude of acid-induced depolarizations in dural TG neurons from pH 6.0-treated mice confirmed the increased function of ASIC previously demonstrated in the chronic nitroglycerin model [14]. We also found that PrNMI reversibly suppressed acid-induced depolarization in dural TG neurons isolated from control (pH 7.4-treated mice (Fig. 5). This finding is in line with previous reports showing that either the brain-permeant cannabinoid WIN 55,212–2 [54] or the peripherally-restricted PrNMI [14] suppress ASIC currents in a CB1R-dependent manner. The latter study also showed that repetitive pretreatment with PrNMI also prevented nitroglycerin-induced increases in expression of phosphorylated PKA, nNOS, and TRPA1 [14]. Here, we demonstrated the loss of PrNMI effectiveness in decreasing ASIC-mediated responses to pH 6.0 in dural TG neurons from pH 6.0-treated mice (Fig. 5). These findings provide a mechanistic explanation for the lack of PrNMI’s posttreatment effectiveness in suppressing the behavioral symptoms of allodynia induced by a mild (pH 7.0) stimulus. The data also suggest that in addition to enhancement of ASIC functional properties, acid-induced sensitization may alter signal transduction at peripheral CB1R sites. These could involve increased internalization of CB1Rs, changes in orthosteric agonist binding pocket, or engagement of alternative second messenger pathways involved in modulation of nociception. For example, in contrast to PrNMI, a novel peripherally-restricted CB1R selective agonist, VIP36, developed through cryptic pocket targeting and biased agonist design, was recently reported to be effective as posttreatment in the chronic nitroglycerin model [44]. This suggests the possibility that CB1R signaling in sensitized nociceptors may respond differently to conventional versus next-generation agonists [44]. Therefore, evaluating novel CB1R ligands such as VIP36 in the acid-induced migraine-like pain model may help elucidating the relationship between GPCR bias and acid-induced sensitization of TG neurons. Alternatively, loss of PrNMI’s effectiveness may involve a switch from PKA to PKC signaling. Among the PKC isoforms, PKCδ and PKCε have been implicated in the pathophysiology of migraine. Both PKCδ and PKCε have been shown to contribute to nitroglycerin-induced mechanical allodynia [55, 56]. Notably, whereas PKCδ facilitates the persistence of migraine-like headache, PKCε does not appear to play a significant role in this process [55]. Collectively, these results provide mechanistic insight into the contribution of peripheral signaling pathways to pain generation under acidic conditions.

### Study limitations

In this model, acidic stimulation induces pain and latent sensitization; however, it remains unclear how such acidic changes occur in meningeal afferent fibers during migraine in humans. Nonsurgical supradural administration of sensitizing and analgesic agents was limited to young mice in which the sutures at bregma remain open. Consequently, long-term chronic studies are not possible in this model. Although PrNMI loses its efficacy once sensitization has been established, investigating the mechanisms underlying is technically challenging because analyzing second messenger signaling in a single-cell–specific manner after sensitization is difficult. In the present experiments, changes in resting potential, action potential properties, cannabinoid receptor, and ASIC-mediated responses were examined in sensitized afferents. However, acid-mediated sensitization of dural afferents could affect the function of numerous other voltage and ligand-gated ion channels, hence, more specific analyses are warranted.

### Conclusions

Our findings indicate that a peripherally-restricted cannabinoid agonist, PrNMI, prevents acid-induced sensitization and allodynia by activating peripheral neuronal CB1Rs and non-neuronal CB2Rs. CB2R activation alone is insufficient, and once dural nociceptors are sensitized, combined CB1R/CB2R activation loses efficacy. Identification of the molecular events that mediate dural nociceptor sensitization should facilitate development of novel targeted treatments that effectively prevent the chronification of migraine episodes.

## Supplementary Material

This is a list of supplementary files associated with this preprint. Click to download.

- GraphicalAbstract.jpg

## Data Availability

All data generated and analyzed during this study are included in this article.
